# ATRX immunostaining predicts IDH and H3F3A status in gliomas

**DOI:** 10.1186/s40478-016-0331-6

**Published:** 2016-06-16

**Authors:** Azadeh Ebrahimi, Marco Skardelly, Irina Bonzheim, Ines Ott, Helmut Mühleisen, Franziska Eckert, Ghazaleh Tabatabai, Jens Schittenhelm

**Affiliations:** Department of Neuropathology, Institute of Pathology and Neuropathology, University Hospital of Tuebingen, Eberhard Karls University of Tuebingen, Calwerstr. 3, D-72076 Tuebingen, Germany; Department of Neurosurgery, University Hospital of Tuebingen, Eberhard Karls University Tuebingen, Tuebingen, 72076 Germany; Department of Pathology, Institute of Pathology and Neuropathology, University Hospital of Tuebingen, Eberhard Karls University of Tuebingen, Tuebingen, 72076 Germany; Department of Pathology, Institute of Pathology and Neuropathology, Ludwigsburg Hospital, Ludwigsburg, 71640 Germany; Department of Radiation Oncology, University Hospital of Tuebingen, Eberhard Karls University of Tuebingen, Tuebingen, 72076 Germany; Interdisciplinary Division of Neurooncology, Departments of Vascular Neurology, Neurosurgery University Hospital of Tuebingen, Eberhard Karls University of Tuebingen, Tuebingen, 72076 Germany; Laboratory for Clinical and Experimental Neurooncology, Hertie-Institute for Clinical Brain Research, Tuebingen, Germany; Center for Personalized Medicine, Eberhard Karls University of Tuebingen, Tuebingen, Germany; German Consortium for Translational Cancer Research (DKTK), DKFZ partner site, Tuebingen, Germany; Center for CNS Tumors, Comprehensive Cancer Center Tuebingen-Stuttgart, University Hospital of Tuebingen, Eberhard Karls University of Tuebingen, Tuebingen, Germany

**Keywords:** Glioma, ATRX, IDH, H3F3A, Astrocytoma, Oligodendroglioma

## Abstract

Gliomas are the most frequent intraaxial CNS neoplasms with a heterogeneous molecular background. Recent studies on diffuse gliomas have shown frequent alterations in the genes involved in chromatin remodelling pathways such as α-thalassemia/mental-retardation-syndrome-X-linked gene (ATRX). Yet, the reliability of ATRX in predicting isocitrate dehydrogenase (IDH) and H3 histone, family 3A (H3F3A) mutations in gliomas, is unclear.

We analysed the ATRX expression status by immunohistochemistry, in a large series of 1064 gliomas and analysed the results in correlation to IDH, H3F3A and loss of heterozygosity (LOH) 1p/19q status in these tumors. We also investigated the prognostic potential of ATRX concerning the clinical outcome of patients with diffuse gliomas.

According to our results, loss of nuclear ATRX expression was accompanied with an astrocytic tumor lineage and a younger age of onset. ATRX loss in astrocytomas was also strongly associated with IDH1/2 and H3F3A mutation (*p* < 0.0001). Among 196 glial tumors with nuclear ATRX loss, 173 (89 %) had an IDH1 or IDH2 mutation. Among the remaining 23 cases (11 %) with ATRX loss and IDH wild type status, 7 cases had a H3F3A G34R mutation (3 %) and 2 cases had a H3F3A K27M mutation (1 %). ATRX retention in IDH1/2 mutant tumors was strongly associated with LOH 1p/19q and oligodendroglioma histology (*p* < 0.0001). We also confirmed the significant prognostic role of ATRX. Diffuse gliomas with ATRX loss (*n* = 137, median 1413 days, 95 % CI: 1065–1860 days) revealed a significantly better clinical outcome compared with tumors with ATRX retention (*n* = 335, median: 609, 95 % CI: 539–760 days, HR = 1.81, *p* < 0.0001).

In conclusion, ATRX is a potential marker for prediction of IDH/H3F3A mutations and substratification of diffuse gliomas into survival relevant tumor groups. Such classification is of great importance for further clinical decision making especially concerning the therapeutic options available for diffuse gliomas.

**Electronic supplementary material:**

The online version of this article (doi:10.1186/s40478-016-0331-6) contains supplementary material, which is available to authorized users.

## Introduction

Gliomas, including astrocytoma and oligodendroglioma are the most frequent primary intraaxial neoplasms. Diffuse astrocytomas are characterized by invasive growth and may progress to glioblastomas through acquisition of additional mutations [1]. Many of these tumors have tumor-promoting mutations in the isocitrate dehydrogenase (IDH) 1 and/or 2 genes [2, 3]. Presence of IDH mutations is associated with a better overall outcome in astrocytic and oligodendroglial neoplasms compared to IDH wild-type tumors [4]. The intratumoral genetic heterogeneity makes it difficult to identify the main oncogenic drivers in gliomas [5]. The presence of similar Tp53 point mutations in low-grade diffuse astrocytomas and secondary glioblastomas suggests that they play a crucial role in astrocytic differentiation [6], whereas 1p19q co-deletions (loss of heterozygosis, LOH) that are present in up to 80 % of oligodendrogliomas, promote an oligodendroglial tumor differentiation [7]. Frequent alterations in the genes involved chromatin remodelling pathways in gliomas, e.g., recurrent mutations in H3 histone, family 3A (H3F3A) and α-thalassemia/mental-retardation-syndrome-X-linked gene (ATRX), a chromatin modifier, have been identified in paediatric gliomas [8]. ATRX alterations are frequent and, according to the current knowledge, are associated with astrocytic tumors carrying additional IDH1/2 and TP53 mutations [9, 10]. Missense and truncating mutations of ATRX gene lead to loss of expression in gliomas [8, 9] and correlates with better clinical outcome in a subset of IDH1 mutant tumors [11].

The reliability of ATRX loss in predicting IDH 1/2 and H3F3A mutations in gliomas has not been investigated extensively so far. In this study, using immunohistochemistry, we analysed the ATRX expression status in a large series of gliomas in order to evaluate 1) the predictive potential of ATRX for IDH1/2 and H3F3A mutations and LOH 1p/19q status, 2) the optimal interobserver-reliant cut-off point for ATRX loss. We also investigated the prognostic potential of ATRX in our cohort in correlation to the relevant clinicoclinico-pathological parameters (survival, age, location, gender, recurrence and histologic appearance).

## Material and methods

### Tissue samples

One thousand sixty-four brain tumor samples were enrolled in this retrospective study. The samples were obtained from patients undergoing surgery for astrocytic, oligodendroglial and ependymal tumours between 2000 and 2015 in the Department of Neurosurgery at the University hospital of Tuebingen. In order to optimize the sampling and avoid any age differences between the primary and secondary glioblastomas occurring in patients who are usually 15 years younger [1], the glioblastoma samples were age-matched (Table 1). The study was authorized by the Tübingen University ethics board (permission number 681/2013BO2). Histological diagnosis and grading for each tumor sample was performed according to the current WHO classification system by at least two experienced neuropathologists [12]. Additional integrated diagnosis (ie. combination of histology with molecular data) was performed as outlined in the upcoming WHO revision of 2016 [13].

**Table 1:** Epidemiological data of samples

Tumor	WHO grade	Samples (n)	Recurrence (n)	Sex (F/M)	Age (mean, range)
Diffuse Astrocytoma		284	43	120/164	45 (5–81)
- Low grade (WHO II)	II	136	14	61/75	43 (9–77)
- Anaplastic (WHO III)	III	148	29	59/89	47 (5–81)
Oligoastrocytoma		71	23	29/42	43 (5–79)
- Low grade (WHO II)	II	38	12	15/23	43 (5–79)
- Anaplastic (WHO III)	III	33	11	14/19	43 (21–71)
Oligodendroglioma		83	32	44/39	48 (24–81)
- Low grade (WHO II)	II	35	7	16/19	46 (24–81)
- Anaplastic (WHO III)	III	48	25	28/20	50 (20–79)
Glioblastoma	IV	415	57	169/246	57 (4–87)
- Primary	IV	364	60	145/219	58 (4–87)
- Secondary	IV	30	30	12/18	46 (26–68)
- With oligodendroglial component	IV	13	3	3/10	61 (45–82)
- With PNET component	IV	1	0	0/1	54
- Gliosarcoma	IV	7	2	7/0	66 (53–75)
Ependymoma		112	25	50/62	41 (2–82)
- Low grade (WHO II)	II	51	4	27/24	47 (2–82)
- Anaplastic (WHO III)	III	26	11	11/15	35 (2–78)
- Myxopapillary	I	24	8	7/17	36 (17–75)
- Subependymoma	I	11	2	5/6	55 (44–75)
Pilocytic astrocytoma	I	74	15	41/33	22 (2–78)
- Pilomyxoid (WHO II)	II	2	1	1/1	8 (2–14)
- Anaplastic (WHO III)	III	2	1	1/1	25 (24–25)

### Tissue microarrays and Immunohistochemistry

Sufficient tumor content was available in 1043 of 1064 samples. Tissue microarrays (TMA) were provided from 613 out of 1043 tumors (59 %, Table 1), and all remaining tumors were analysed on full slides. For TMAs, using a TMA machine (Beecher Instruments, Inc., Sun Prairie, WI, USA), two cylindrical representative tissue core biopsies of 1000 μm diameter were punched from each paraffin donor block and transferred to the pre-punched holes on recipient paraffin blocks at defined array coordinates. After a short sealing of the recipient blocks at 37 °C, the TMA blocks were cut with a microtome (Microm International, Walldorf, Germany) into 4 μM thick sections, and mounted on SuperFrost Plus slides. Afterwards, the slides were stained with hematoxylin and eosin and reviewed for adequate tumor representation in the individual cores. ATRX and IDH1 R132H immunostains were performed on an automated immunohistochemistry system (BenchMark, Ventana Medical Systems, Strasbourg, France), as previously described [4]. Settings for the ATRX OptiView method were: Cell Conditioner 1 pretreatment for 40mins, primary antibody incubation for 20 min at 42 °C, antibody dilution 1:400, counterstaining with hematoxylin. All reagents were provided from Ventana Medical Systems, Strasbourg, France, rabbit anti-ATRX 0.1 mg/ml from Sigma St. Louis, MO, USA.

### Evaluation and quantification of ATRX staining

**Fig. 1:**
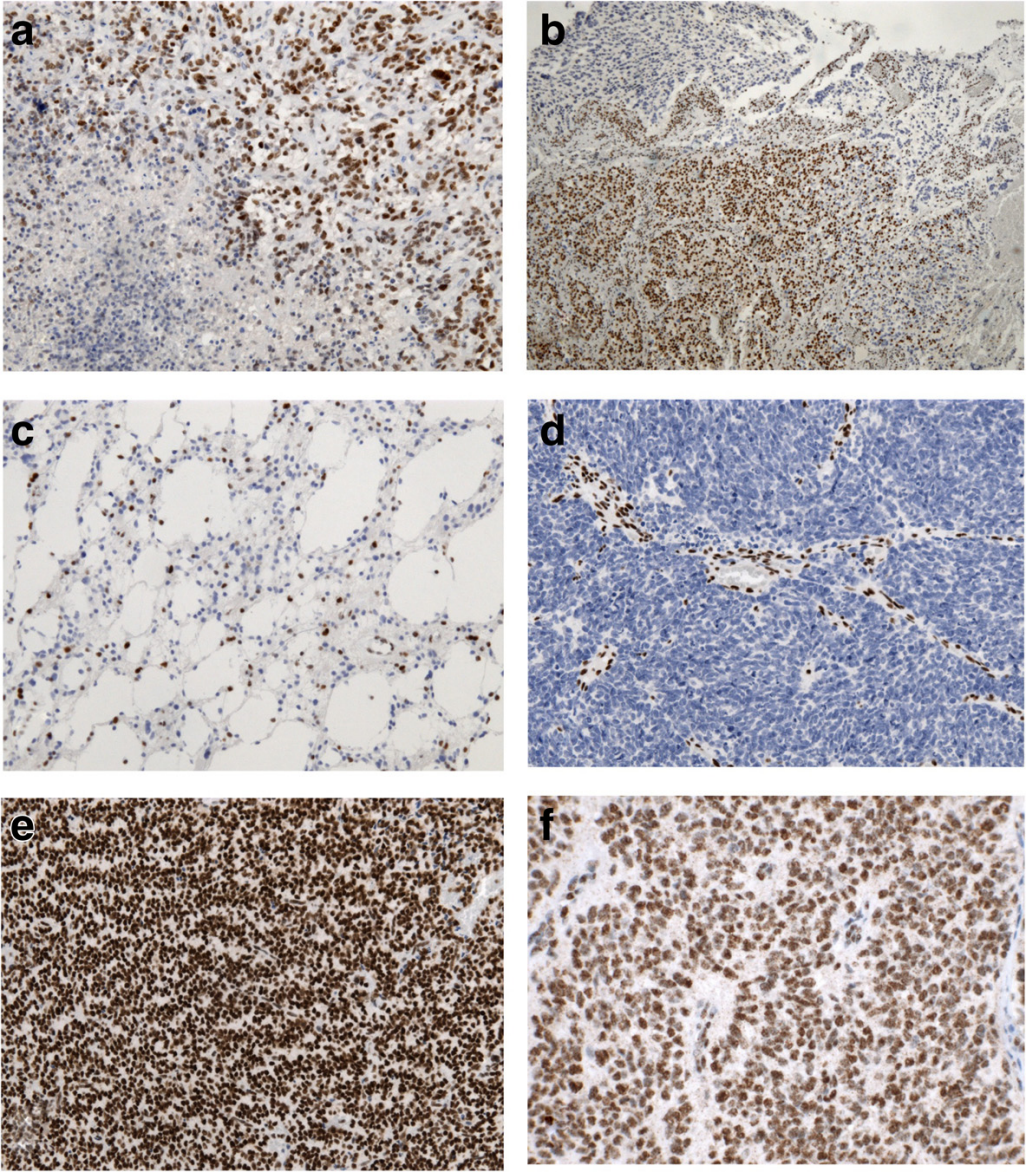
ATRX immunohistochemistry: **a** loss of immunoreactivity in necrotic/perinecrotic tumor areas, **b** Ambiguous ATRX status; areas with clear ATRX retention bordering areas with nuclear ATRX loss. **c** Diffuse astrocytoma grade II with ATRX loss, **d** Anaplastic Astrocytoma grade III with ATRX loss, **e** Oligodendroglioma grade II with ATRX retention, **f** Anaplastic Ependymoma with ATRX retention (**f** magnification ×200, **a**, **c**–**e** magnification ×100, **b** magnification ×40)

TMAs and full slides were independently evaluated by at least two different neuropathologists. All TMAs were screened for internal positive controls such as endothelial cells and/or trapped cortical neurons. Twenty-four sample punches from four representative tissue microarrays were then selected and ATRX re-staining was performed in whole tissue sections of the same tumor for validation of the results. In accordance with previous studies samples were considered positive when the tumor cells showed nuclear immune reactivity for ATRX and were considered negative, when the tumor cells showed no nuclear positivity in the presence of accurate internal positive controls [9, 11, 14]. Nuclear staining was not evaluated in areas bordering geographic necrosis and/or apoptosis (Fig. 1a) as well as in borders of very large specimens where the staining was reduced or absent. Samples with insufficient tumor content, extensive necrosis in more than 90 % of TMA core and large hematoma deposits on TMA cores were excluded. The samples with ATRX negativity in internal positive controls and samples with ambiguous results (displaying clear heterogeneity of staining pattern within the same tumor with areas showing partially preserved and partially absent nuclear ATRX, Fig. 1b) were excluded as well. The percentage of positive nuclei among the total stained tumor area was recorded and data was collected in 866 cases. Based on this data, the tumors where then divided into two groups: with nuclear ATRX loss and with nuclear ATRX retention. A cut-off point of 10 % was considered for evaluation of presence or absence of nuclear ATRX.

### IDH and H3F3A mutation and MGMT promoter methylation analysis

The IDH1 mutational status was first determined immunohistochemically by a mutation-specific monoclonal antibody against the IDH1 R132H mutation (dianova, Hamburg, Germany) as previously described [4]. Diffuse glioma samples lacking the R132H mutation were further examined by direct sequencing of the relevant exon for IDH1 and 2 mutations. Using a BlackPREP FFPE kit (Analytik Jena, Germany), DNA was extracted from the tumor tissue according to the manufacturer’s instructions. Tissue was selected from regions on paraffin blocks that presented sufficient tumor content in microscopy. The region around IDH1 codon 132 was amplified as previously described [15]. PCR for IDH2 hotspot mutation analysis was performed using the primer pair IDH2 up 5’-CTGTCCTCACAGAGTTCAAGC-3’ and IDH2 lo 5’-CTGTCCTCACAGAGTTCAAGC-3’. Immunohistochemistry for H3F3A K27M was performed using rabbit anti human Histone H3 (K27M mutant) antibody (dilution 1:1500, incubation 32 min, Merck Millipore, Billeria, MA, USA). Sequencing for H3F3A was performed, as follows: PCR products were purified (AMPure, Beckman Coulter, Brea, CA, USA) and aliquots of 7 μl were used for the sequencing reaction with 1 μM of the universal M13 sequencing primer and 2 μl of GenomeLab DTCS-Quick Start Kit (Beckman Coulter, Brea, CA, USA) in a final volume of 10 μl according to the manufacturers protocol. Sequencing reactions were purified (CleanSEQ, Beckman Coulter, Brea, CA, USA) and analyzed in a GenomeLab GeXP Genetic Analysis System and evaluated by the GenomeLab GeXP software 10.2 (Beckman Coulter, Brea, CA, USA) to determine the mutation status. Loss of heterozygosity (LOH 1p/19q) was examined using 5 tetranucleotide markers for each chromosomal region and analysed on synthetic high resolution Spreadex gels (Elchrom Scientific, Germany) as previously described [16]. Allele signal intensity of each tumor sample was always compared with the corresponding allele band of the blood control sample taken from the same patient. Bisulfite treatment of DNA and analysis for MGMT promoter methylation was performed by methylation-specific PCR (MSP-PCR) in WHO grade III and IV tumors as previously described using methylated and unmethylated controls [17].

### Statistical analysis

Quantitative and statistical analyses were performed using JMP 7.0 (SAS Institute, Cary, NJ, USA). For correlation analyses we performed unpaired, two-tailed Student’s *t*-test and the Fisher’s exact test to identify possible significant associations or differences between two pairs. For multiple comparison, ANOVA (one- or two-way) or Kruskal–Wallis testing was used. To assess the potential interobserver variability, we performed a Pearsons Chi-square test on the data collected by the two neuropathologists and calculated Cohen's kappa coefficient for agreement [18]. Kaplan-Meier test was performed for survival analysis in the astrocytoma, oligodendroglioma and glioblastoma cohorts (*n* = 798). Ependymoma and pilocytic astrocytoma were excluded from survival analysis. Univariate analyzes of the different variables were obtained with 95 % confidence intervals (CIs) followed by Cox multivariate logistic regression analyses and hazard ratio (HR) calculation. A *p*-value < 0.05 was considered as significant.

## Results

### Loss of nuclear ATRX expression is accompanied with an astrocytic tumor lineage and a younger age of onset

Loss of nuclear ATRX in tumor cells was found in 210 out of 885 (24 %) of the tumors (Mean positive nuclei: 7.9 %, 95 % CI: 5.6–10.1). ATRX retention was observed in 675 out of 885 (76 %) samples (Mean positive nuclei: 78.9 %, 95 % CI: 77.6–80.1, *p* < 0.0001). Data from both neuropathologists were highly concordant (Pearsons Chi-square: *p* < 0.0001; Cohens kappa agreement: 0.18; 2^nd^ dataset ATRX loss: mean: 12.1 %; 95 % CI: 6.2–17.9; ATRX retention: mean 67.1 %; 95 % CI: 64.4–69.8). Detailed results on ATRX status are listed in Table 2.

**Table 2:** Results of ATRX and IDH mutation status stratified by histological diagnosis

Tumor	WHO Grade	ATRX loss/retention (n)^a^	IDH mutated/WT (n)^a^	IDH mutant & ATRX loss/retention (n)^a^	H3F3A mutant & ATRX loss/retention (n)^a^
Diffuse Astrocytoma		136/120 (53 %)	156/113 (42 %)	116/20 (85 %)	
- Low grade	II	72/49 (59 %)	86/44 (66 %)	62/12 (84 %)	
- Anaplastic	III	64/71 (47 %)	70/69 (50 %)	52/10 (84 %)	4/0 (100 %)
Oligoastrocytoma		23/48 (32 %)	66/6 (92 %)	23/42 (35 %)	
- Low grade	II	8/31 (21 %)	36/3 (92 %)	8/28 (23 %)	
- Anaplastic	III	15/17 (47 %)	30/3 (91 %)	15/14 (52 %)	
Oligodendroglioma		1/79 (1 %)	81/2 (98 %)	1/77 (1 %)	
- Low grade	II	0/33 (0 %)	35/0 (100 %)	0/33 (0 %)	
- Anaplastic	III	1/46 (2 %)	46/2 (96 %)	1/44 (2 %)	
Glioblastoma	IV	47/260 (15 %)	38/295 (11 %)	33/9 (79 %)	5/7 (42 %)
- Primary	IV	27/215 (11 %)	19/224 (7 %)	17/3 (89 %)	5/7 (42 %)
- Secondary	IV	16/14 (53 %)	19/11 (63 %)	14/5 (78 %)	
- With oligodendroglial component	IV	3/10 (23 %)	4/8 (33 %)	3/1 (75 %)	
- With PNET component	IV	0/1 (0 %)	0/1 (0 %)		
- Gliosarcoma	IV	0/6 (0 %)	0/6 (0 %)		
Ependymoma		0/93 (0 %)	0/98 (0 %)		
- Low grade	II	0/43 (0 %)	0/41 (0 %)		
- Anaplastic	III	0/23 (0 %)	0/23 (0 %)		
- Myxopapillary	I	0/18 (0 %)	0/24 (0 %)		
- Subependymoma	I	0/9 (0 %)	0/10 (0 %)		
Pilocytic astrocytoma	I	0/57 (0 %)	0/57 (0 %)		
- Pilomyxoid	II	0/1 (0 %)	0/1 (0 %)		
- Anaplastic	III	1/1 (50 %)	0/2 (0 %)		

^a^Values in brackets show the percentage of tumors with ATRX loss within the respective sample cohort. For Ependymoma and pilocytic, pilomyxoid and anaplastic pilocytic astrocytoma ony the results of IDH1 (R132)-IHC were available

ATRX loss is significantly associated with tumors of astrocytic lineage including astrocytoma grade II (Fig. 1c), III (Fig. 1d) and glioblastoma grade IV with the exception of pilocytic astrocytomas (*p* < 0.001). Fifty-three percent of astrocytomas grade II and III and 53 % of secondary glioblastomas showed a nuclear ATRX loss. Frequency of ATRX loss in primary glioblastomas (11 %) and oligoastrocytomas (32 %) was markedly lower than in astrocytomas and secondary glioblastoma. Only in one oligodendroglioma (1 %) ATRX was lost, (Fig. 1e). ATRX retention was seen in all ependymal tumors (Fig. 1f) and pilocytic astrocytomas, except for a single case diagnosed as anaplastic pilocytic astrocytoma. ATRX loss was more frequent in tumors emerging from the frontal lobe (*p* < 0.0001, Table 3).

**Table 3:** Results of ATRX expression status stratified by tumor location

Tumor location	ATRX loss/retention (%)	ATRX loss/retention (n)
Frontal lobe	48/29	79/27
Parietal lobe	7/9	12/44
Temporal lobe	26/30	43/139
Occipital lobe	2/4	4/20
Thalamus	1/2	1/8
Intraventricular	1/2	1/10
Brainstem and midbrain	1/2	1/11
Cerebellum	1/5	2/24
Spinal cord	1/10	1/49
Multiple locations	13/6	21/27

Patients with ATRX loss were significantly younger than those with ATRX retention (mean age 36.6 vs. 48.5 years, *p* <0.0001). The differences remained significant after stratification of tumors according to WHO grade (*p* = 0.0104 for WHO II tumors versus *p* < 0.0001 for WHO IV tumors). Within the group of diffuse gliomas diagnosed below the age of 45 years, ATRX loss was observed in 144/278 (52 %) of tumors. ATRX loss was more frequent in secondary glioblastomas compared to age-matched primary glioblastomas (34 %) (*p* = 0.0068). No gender-related differences in ATRX status were observed. ATRX expression status was not significantly different in primary and recurrent tumors (ATRX loss 27 % in primary vs. 28 % in recurrence, *p* = 0.75). Besides, initial interpretations on 24 selected sample punches, chosen for validation of results, could be recalled in the full slides of the same tumors. Percentage of stained nuclei from both stains were assessed and were highly concordant (Pearsons Chi-square: *p* < 0.0001; Cohens kappa agreement: 0.21, Detailed data are available in Additional file 1).

### ATRX loss in astrocytomas is strongly associated with IDH1/2 and H3F3A mutation

**Fig. 2:**
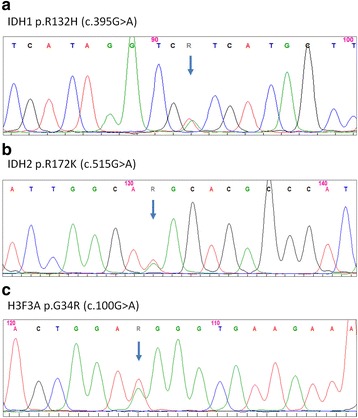
Representative examples of the detected IDH1, IDH2 and H3F3A mutations. **a** Electropherogramm of IDH1 p.R132H (c.395G > A), **b** electropherogramm of IDH2 p.R172K (c.515G > A), **c** electropherogramm of H3F3A p.G34R (c.100G > A). Blue arrow designates the mutation

IDH1/2 mutation analysis was available in 838 samples. 345 out of 838 samples (41 %) revealed an IDH mutation (Table 2). The canonical IDH1 p.R132H mutation was found in 305 samples (88 %). The less common mutations included IDH1 p.R132C in 11 (3.3 %), IDH2 p.R172K in 8 (0.2 %), IDH1 p.R132G in 9 (0.3 %), IDH1 p.R132S in 4 (0.1 %), IDH2 p.R172S in 3 (0.1 %), p.R172M in 3 (0.1 %), IDH p.R132L in one (0.03 %) and IDH2 p.R172W mutation in one (0.03 %) tumors (Fig. 2a, b).

ATRX loss was significantly associated with IDH mutation (*p* < 0.0001). Among 196 tumors with nuclear ATRX loss, 173 (89 %) had an IDH1 or IDH2 mutation (Table 2). Among the remaining 23 cases (11 %) with ATRX loss and IDH wild type (wt) status (10 anaplastic astrocytoma, 13 glioblastoma), 7 cases had a H3F3A p.G34R mutation (3 %, Fig. 2c) and 2 cases had a H3F3A p.K27M mutation (1 %). In 3 cases, a H3F3A wild-type status was confirmed including one case of cerebellar glioblastoma. Upon histological and further molecular re-evaluation, a KIAA1549-BRAF fusion was found in the cerebellar GBM case indicating that this case might be grouped as anaplastic pilocytic astrocytoma. One remaining case was associated with HNPCC syndrome, a condition associated with gliomagenesis [19]. ATRX loss was significantly associated with a H3F3A p.G34R mutation (*p* = 0.0098). Among the IDH1/2 wt tumors without ATRX loss, 7 cases with a H3F3A p.K27M mutation were identified (Table 2). All these tumors fulfilled the location-specific criteria for a K27M-mutant midline glioma. No combined IDH1/2 and H3F3A mutation was found. The mean age of patients with grade III and IV tumors and ATRX loss was significantly lower compared to the tumors with ATRX retention (39 vs 55 years, *p* < .0001).

Patients’ age in tumors with ATRX loss, IDH wt and H3F3A wt was significantly lower than those with the same histological diagnosis and ATRX retention but IDH mutated and/or H3F3A mutated (44 vs. 59 years *p* = 0.0003). The percentage of tumors with ATRX loss and methylated MGMT promotor (26.4 %, 38/144) did not differ from tumors with ATRX retention and MGMT promotor methylation (25.8 %, 48/186, *p* = 0.9).

### ATRX retention in IDH1/2 mutant tumors is strongly associated with LOH 1p/19q

Among 148 tumors with IDH mutation and ATRX retention, 77 cases were diagnosed as oligodendroglioma, 42 as oligoastrocytoma, 20 as astrocytoma and 9 as glioblastoma including 5 secondary glioblastoma. ATRX retention was significantly associated with occurrence of combined LOH 1p/19q (LOH1p/19q; *n* = 71 vs. no LOH1p/19q; *n* = 24, *p* < 0.0001). Interestingly, a single case histologically diagnosed as oligodendroglioma, harbouring an ATRX loss, showed no LOH 1p/19q. In the oligoastrocytomas group, tumors exhibited either ATRX loss (*n* = 10) or LOH 1p/19q (*n* = 24) in a mutually exclusive manner, except for a single case showing combined ATRX loss and LOH 1p/19q.

### ATRX status in tumors determines prognostic relevant subgroup

Among the diffuse glioma group irrespective of WHO grade with available survival data (*n* = 472), tumors with ATRX loss (*n* = 137) revealed a significantly better outcome compared to tumors with ATRX retention (*n* = 335) (median survival: 1413 vs. 609 days, HR = 1.81, 95 % CI = 1.43–2.30, *p* < 0.0001). The expected, well-known, significant univariate differences for IDH mutation, WHO grade and patients age (all *p* < 0.0001) were also present in this cohort (Additional file 2). In multivariate regression analysis of these parameters, age (*p* = 0.0004, HR = 3.63, 95 % CI = 1.77–7.44), WHO grade (*p* < 0.0001, HR = 2.27, 95 % CI = 1.60–3.24) and IDH mutation status (p = 0.045, HR = 1.38, 95 % CI = 1.01–1.89) remained significant parameters, while ATRX (*p* = 0.45, HR = 1.11, 95 % CI = 0.83–1.49 for ATRX retention) was no longer significant for all diffuse glioma combined.

Stratification of the tumors for IDH mutation status irrespective of their underlying histology, showed that the outcome of IDH mutant tumors with ATRX loss (*n* = 115, median survival: 1492 days) was statistically not different from IDH mutant tumors with ATRX retention (*n* = 100, median: 1132 days, *p* = 0.1389). Subset analysis of IDH mutant tumors for WHO grade and histology did not reveal any differences, although a trend to worse outcome was seen in the 15 remaining astrocytomas that are IDH mutated and retain ATRX expression (median 1644 days, *p* = 0.0075, Additional file 2).

**Fig. 3:**
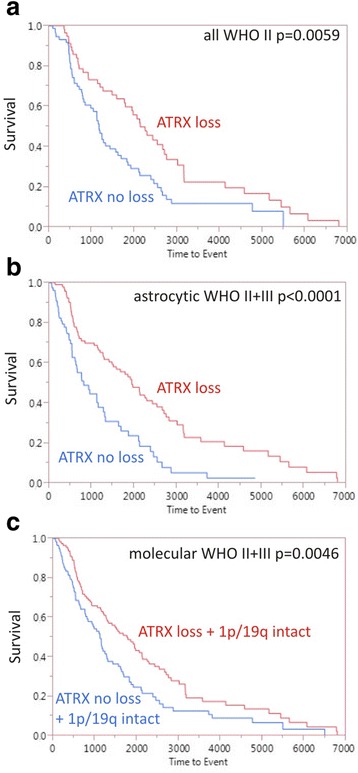
Kaplan-Meier survival plots. **a** Diffuse gliomas of WHO G7rade II separated by ATRX status (*n* = 130); median: 2141 days in ATRX loss group vs. 1175 days in ATRX retention group. **b** Astrocytoma WHO grade II and III grouped (*n* = 176), tumors harbouring ATRX loss (*n* = 96) had a significantly better outcome compared to tumors with ATRX retention (*n* = 80) (median: 1952 vs. 818 days, *p* < 0.0001) **c** Astrocytic, oligodendroglial and mixed tumors without 1p/19q codeletion (*n* = 230) show a significant better outcome when accompanied with ATRX loss than with ATRX retention (median ATRX loss 1769 days vs. ATRX retention 1127 days, *p* = 0.0046)

Next, we controlled tumors for their WHO grades. In univariate analyses, ATRX loss in low grade gliomas was significantly associated with a better survival (Fig. 3a, WHO II: *p* = 0.0059; HR = 1.75, 95CI = 1.17–2.63) but not in high-grade tumors (WHO III: *p* = 0.09; WHO IV: *p* = 0.35).

Among tumors histologically diagnosed as astrocytoma WHO grade II and III combined (*n* = 176), the tumors harbouring ATRX loss (*n* = 96, median: 1952) had a significantly better outcome compared to tumors with ATRX retention (Fig. 3b, *n* = 80, median 818 days, HR = 2.21, 95 % CI = 1.50–3.23, *p* < 0.0001). In multivariate analysis of grade II and III astrocytomas, only IDH mutation (*p* = 0.0469) remained prognostic, but not WHO grade, age and ATRX status.

Finally, molecular substratification of tumors according the WHO 2016 revision (i.e., absence of LOH 1p/19q as the defining marker for oligodendrogliomas) confirmed the prognostic role of ATRX (loss: *n* = 112; median: 1769 days) compared to tumors without ATRX loss (Fig. 3c, *n* = 118; median: 1127 days, HR = 1.56, 95%CI = 1.14–2.13, *p* = 0.0046). Again, multivariate analysis of this cohort indicated presence of IDH mutation as the single prognostic factor (*p* = 0.0022), but not WHO grade, age and ATRX status.

## Discussion

Currently, the WHO classification of brain tumours relies on histopathological and molecular findings. Recent studies indicate that molecular data such as IDH mutation and 1p/19q codeletion have prognostic and predictive significance in gliomas independent of the tumor WHO grade [20, 21]. On the other hand, the prognostic role of WHO grade in IDH mutant tumors is vanishing [22]. In addition, mutations in ATRX were shown to be associated with an alternative lengthening of telomeres (ALT) in gliomas and prognostically relevant in these tumors [11, 23]. The presence of ATRX mutations/ALT phenotype in glial tumors can be visualized through loss of nuclear staining in immunohistochemistry (IHC) [24]. ATRX has been shown to be a potential biomarker in brain tumors [25]. ATRX alterations are closely associated with IDH mutations [22]. The major aim of this study was to determine the predictive role of ATRX for the presence of IDH1/2 or H3F3A mutations in gliomas showing ATRX loss. So far different approaches for definition of ATRX loss have been employed. To our knowledge potential interobserver variability in immunohistochemical evaluation of ATRX and quantification of ATRX loss within the tumor have not been addressed previously. Some authors propose a four-tiered scoring system similar to Her2/neu assessment, ranging from 0 to 3+ [26]. Some neuropathologists suggest a cut-off of 10 % and more ATRX positive cells in tumor serving as ATRX retention [11, 25], while others describe a direct morphological approach with endothelial and pre-existing glial cells serving as internal positive control [27]. In our study we first evaluated the interobserver variability in staining interpretation. We obtained an almost perfect agreement in interpretation of tumors as “ATRX loss” or “ATRX retention” using a 10 % cut-off point for stained nuclei, while direct scoring of the percentage of positive/negative nuclei showed only a moderate agreement between the two observers, mainly due to differences in interpretation of perinecrotic, unstained areas in glioblastoma. Our validation test also indicates that ATRX has a relatively homogenous pattern so that in most cases a single TMA punch (1000 μm diameter) from the viable tumor center is sufficient for the ATRX status interpretation.

Our data shows that ATRX is a helpful marker to detect important coincidental molecular alterations in brain tumors. Among 196 tumors with ATRX loss, we identified 182 cases with either IDH1/2 or H3F3A mutation in a mutually exclusive manner (93 %). Recent studies have shown a coincidence of ATRX loss and IDH mutations in a subset of diffuse gliomas [28, 29]. Concordantly, our data revealed a high correlation of ATRX loss with IDH mutations including canonical and non-canonical ones in 89 % of the cases. In our study the majority of the remaining cases with ATRX loss and IDH wild type, exhibited either H3F3A G34 or K27 mutations, indicating common changes in the ATP-dependent helicase methylation patterns across a wide spectrum of paediatric and early adulthood glioblastomas [30]. In a previous study overlap of ATRX loss and H3F3A mutations were seen in 9 pediatric IDH wildtype glioblastomas [31]. Usually molecular IDH mutation analysis is not performed in primary brain tumors above 55 years, due to the expected low frequency of mutations. In our cohort, ATRX loss was seen in 26 patients above 55 years and an IDH mutation was found in 22 cases. According to this data, tumors with loss of nuclear ATRX and immunohistochemically negative IDH1 R132H should undergo additional sequencing for IDH1/2 and H3F3A irrespective of the patient’s age, as these markers are of great importance for further classification of tumor and choosing the appropriate therapeutic regimen. In our cohort, tumors in the frontal lobe show the highest proportion of ATRX-deficient tumors. This is further corroborated by the relatively high frequency of seizures in the frontal lobe attributed to IDH-mutant gliomas [32]. Nevertheless, ATRX retention does not necessarily exclude IDH mutation itself. In our study almost all tumors with oligodendroglial molecular signature (LOH1p/19q, IDH-mutated) were tumors with ATRX retention. Likewise a post-hoc analysis of the EORTC 26951 oligodendroglioma cohort revealed only one case with ATRX loss among 49 tumors with 1p/19q codeletion [33]. In a different study with only grade II diffuse gliomas, none of the 12 IDH mutated, 1p/19q codeleted tumors had a nuclear ATRX loss [34]. In our cohort, approximately half (51 %) of the IDH-mutated tumors with ATRX retention, had a LOH 1p/19q. Previous studies have tried to predict 1p/19q status in IDH mutated tumors based on a solely ATRX examination [35]. However, according to our results, there are IDH-mutated tumors without combined ATRX loss and 1p/19q codeletion that will be misdiagnosed according to the proposed predictive diagnostic algorithm. As a result, to be able to reliably classify diffuse gliomas, both analyses (ATRX, IDH) should be performed in these tumors, in case they are not already classified as immunohistochemically IDH1 R132H-negative primary glioblastomas. Additionally, like previous studies, our results as an independent patient cohort confirms that oligoastrocytoma can be reclassified on a molecular basis either as molecular astrocytoma or oligodendroglioma and this justifies the removal of oligoastrocytoma group in the current WHO 2016 revision [36]. In our cohort, all H3F3A G34-mutated tumors could have been detected by ATRX loss, while only 2 of 9 K27M-mutated tumors showed absence of nuclear ATRX. Because of their unusual localisation (thalamic/brainstem), these tumors are in potential candidates to apply the diagnostic screening for K27-mutant glioma of the midline. Based on our result, considering the ATRX retention in all ependymal tumors and grade I pilocytic astrocytomas, presence of ATRX loss in such tumors should prompt a histopathological reevaluation of the tumor. The presence of ATRX loss in an anaplastic pilocytic astrocytoma and a cerebellar glioblastoma harbouring a BRAF fusion, more typical for anaplastic pilocytic astrocytoma, in our series remains to be elucidated. Currently, studies on larger series of anaplastic pilocytic astrocytomas are underway.

Besides, we observed a significant prognostic role for ATRX in diffuse astrocytomas. A previous study with relatively small astrocytoma sample was not able to identify a prognostic role for ATRX loss [33]. In a different study, anaplastic gliomas were classified based on ATRX, CIC, FUBP1 and IDH status and patients with ATRX and IDH mutated tumors showed a significantly longer overall survival compared to patients with mutated IDH and wild type ATRX tumors [23]. In another study, samples were retrospectively grouped according to ATRX, IDH and 1p/19q status, where astrocytomas with ATRX loss revealed a significantly better prognosis than those with ATRX retention (median time to treatment failure 55.6 vs. 31.8 months) [11]. However, multivariate regression analysis of the whole samples irrespective of tumor subtype found no significant role for ATRX, due to the superior clinical course of oligodendrogliomas that mainly show a nuclear ATRX retention. Noteably, WHO grade in this cohorts were also no longer significant, confirming the previously observed grading problem for IDH mutated tumors [22]. Limitations of this study include the retrospective nature, the divergent treatment and the different age groups that may confound the prognostic impact. Furthermore, prospective studies incorporating all clinically relevant molecular markers together are needed to evaluate the optimal treatment for these molecular subgroups.

## Conclusions

Our data indicates, that nuclear ATRX loss in glioma is a helpful marker for predicting both, IDH1/2 and H3F3A mutations status in tumors. We were able to confirm that ATRX has a prognostic value with better outcome in astrocytic tumors. Combining the current data and the results from previous studies [11, 23, 27] ATRX is a potential marker for substratification of grade II and grade III astrocytomas, oligodendrogliomas, and mixed oligoastrocytomas into following survival relevant tumor groups recapitulating that scheme favoured by The Cancer Genome Atlas [37].

- Group 1: with IDH mutation, no ATRX loss and 1p/19q co-deletion. Most of these tumors histologically resemble the oligodendrogliomas and oligoastrocytomas and have the best prognosis.
- Group 2: with IDH mutation, ATRX loss and without 1p/19q co-deletion. These tumors are usually classified as astrocytomas or oligoastrocytomas and show an intermediate prognosis.
- Group 3: with IDH wild type and ATRX retention. Recently published data indicate that within this group, tumors carrying additional TERT hotspot mutations or chromosome 10q gain, might show a poor prognosis and seem to behave clinically like glioblastomas [38].

There are still a few numbers of tumors that exhibit a loss of nuclear ATRX in the absence of IDH and H3F3A mutations as well as a small fraction of IDH-mutated tumors without both ATRX mutation and 1p/19 co-deletion that cannot be classified according to our proposed algorithm. This indicates that other molecular pathways might be involved in pathophysiology of these tumor subsets [31]. Further studies are needed to reveal further possible molecular drivers in these tumors.

## Additional files

Additional file 1: Overview of ATRX staining results in tissue microarrays and full slides in the same tumors. (XLS 36 kb) Additional file 2: Additional statistical data for IDH, MGMT, WHO, 1p/19q codeletion, age and diagnosis. (XLSX 11 kb)
